# Comment on “Interrelationship between Sleep and Exercise: A Systematic Review”

**DOI:** 10.1155/2017/7301676

**Published:** 2017-08-20

**Authors:** Mohamed A. Mejri, Omar Hammouda, Amel Tayech, Narimen Yousfi, Anis Chaouachi, Nizar Souissi

**Affiliations:** ^1^Research Laboratory “Sport Performance Optimization”, National Center of Medicine and Sciences in Sport (CNMSS), Tunis, Tunisia; ^2^Faculty of Science, Carthage University, Bizerte, Tunisia; ^3^High Institute of Sport and Physical Education, Ksar-Saïd, Manouba University, Tunis, Tunisia; ^4^Laboratoire CeRSM (EA 2931), Equipe de Physiologie, Biomécanique et Imagerie du Mouvement, UFR STAPS, Université Paris Ouest Nanterre La Défense, Nanterre, France; ^5^National Observatory of Sports, Tunis, Tunisia

We have read with interest the article from Dolezal et al. [1], entitled “Interrelationship between Sleep and Exercise: A Systematic Review,” which highlighted the links between sleep, physical exercise, and health. As authors of the articles [55, 56] cited in the above manuscript [1], we would like to correct information in Section 4.4, “Exercise and Sleep in Special Populations”: “Several studies reported that one night of sleep deprivation can result in metabolic irregularities, such as decreased plasma lactate concentration as well as increased creatine phosphokinase and myoglobin levels, after a bout of exercise the following morning [55, 56].” Please note the exercise was not done the morning after the night of sleep deprivation. Rather, it was done the following evening (i.e., at 17:00 h) [55, 56].

This methodological detail is very important, given that in our previous study [2] we did not find any effect of one night of sleep deprivation on performance or plasma lactate levels during a bout of exercise performed the following morning (i.e., at 07:00 h). On the other hand, we have found that one-night sleep deprivation did not affect the basal physiological responses (i.e., heart rate, arterial oxygen saturation, and systolic and diastolic blood pressure) and biomarkers of lipid profile (i.e., total cholesterol, high-density lipoprotein cholesterol, low-density lipoprotein cholesterol, and triglyceride) [3]. However, basal cardiac damage biomarkers were significantly increased in this study [3].
